# Expression of Rod-Derived Cone Viability Factor: Dual Role of CRX in Regulating Promoter Activity and Cell-Type Specificity

**DOI:** 10.1371/journal.pone.0013075

**Published:** 2010-10-07

**Authors:** Sophie Lambard, Sacha Reichman, Cynthia Berlinicke, Marie-Laure Niepon, Olivier Goureau, José-Alain Sahel, Thierry Léveillard, Donald J. Zack

**Affiliations:** 1 Department of Genetics, INSERM, U968, Paris, France; 2 INSERM UPMC Univ Paris 06, UMR_S 968, Institut de la Vision, Paris, France; 3 CNRS, UMR_7210, Paris, France; 4 Department of Ophthalmology, Johns Hopkins University School of Medicine, Baltimore, Maryland, United States of America; 5 Department of Molecular Biology and Genetics, Department of Neuroscience, and Institute of Genetic Medicine, Johns Hopkins University School of Medicine, Baltimore, Maryland, United States of America; National Institute on Aging, National Institutes of Health, United States of America

## Abstract

**Background:**

RdCVF and RdCVF2, encoded by the nucleoredoxin-like genes *NXNL1* and *NXNL2*, are trophic factors with therapeutic potential that are involved in cone photoreceptor survival. Studying how their expression is regulated in the retina has implications for understanding both their activity and the mechanisms determining cell-type specificity within the retina.

**Methodology/Principal Findings:**

In order to define and characterize their promoters, a series of luciferase/GFP reporter constructs that contain various fragments of the 5′-upstream region of each gene, both murine and human, were tested in photoreceptor-like and non-photoreceptor cell lines and also in a biologically more relevant mouse retinal explant system. For *NXNL1*, 5′-deletion analysis identified the human −205/+57 bp and murine −351/+51 bp regions as having promoter activity. Moreover, in the retinal explants these constructs drove expression specifically to photoreceptor cells. For *NXNL2*, the human −393/+27 bp and murine −195/+70 bp regions were found to be sufficient for promoter activity. However, despite the fact that endogenous *NXNL2* expression is photoreceptor-specific within the retina, neither of these DNA sequences nor larger upstream regions demonstrated photoreceptor-specific expression. Further analysis showed that a 79 bp *NXNL2* positive regulatory sequence (−393 to 315 bp) combined with a 134 bp inactive minimal *NXNL1* promoter fragment (−77 to +57 bp) was able to drive photoreceptor-specific expression, suggesting that the minimal *NXNL1* fragment contains latent elements that encode cell-type specificity. Finally, based on bioinformatic analysis that suggested the importance of a CRX binding site within the minimal *NXNL1* fragment, we found by mutation analysis that, depending on the context, the CRX site can play a dual role.

**Conclusions/Significance:**

The regulation of the Nucleoredoxin-like genes involves a CRX responsive element that can act as both as a positive regulator of promoter activity and as a modulator of cell-type specificity.

## Introduction

Retinitis pigmentosa (RP) is a genetically and clinically heterogeneous group of inherited retinal degenerative diseases that causes severe visual impairment in as many as 2 million patients worldwide [1], [2]. Most identified forms of RP are caused by mutations in genes whose expression is enriched in or restricted to rod photoreceptor cells [3], [4]. The loss of rods in RP is generally followed by a secondary degeneration of cone photoreceptors [5], [6]. It is this secondary loss of cones that is most significant clinically because cones are required for high acuity, color, and most daylight vision. In the vast majority of cases, RP and related forms of retinal degeneration are untreatable, and all that can be offered to patients are supportive measures.

Given the clinical importance of cone loss in retinal degenerative disease, in our effort to develop broad-based therapies for RP that go beyond single gene replacement, we have been studying the mechanisms of cone cell loss. We and our colleagues have demonstrated that rods secrete factors that promote cone survival, reviewed in [7]. Furthermore, through expression and homology cloning approaches, we have identified a novel family of rod-secreted factors that are trophic for cones. These factors, Rod-Derived Cone Viability Factor (RdCVF) and RdCVF2, are encoded, respectively, by the nucleoredoxin-like genes *NXNL1* and *NXNL2* [8], [9]. The cone-directed protective activity of retinal explant conditioned media is significantly reduced after immunodepletion of RdCVF, suggesting that a major part of retinal derived cone survival activity *in vivo* is due to RdCVF [8]. The degeneration of rods in the *rd1* mouse model of RP results in the loss of expression of RdCVF by rods as well as by bipolar cells at the onset of cone degeneration [8], [10]. The potential of RdCVF administration for promoting survival of the central vision attributable to cones in patients suffering from RP is well illustrated by the finding that injection of RdCVF protein in the P23H transgenic rat model of dominant RP both slows down secondary cone loss and, more importantly, preserves cone function as measured by electroretinography [11].

Both RdCVF and RdCVF2 are encoded by an unspliced transcript from their respective genes. *NXNL1* and *2* also express a spliced mRNA that encodes the protein products RdCVFL and RdCVF2L, which unlike RdCVF and 2 contain an entire thioredioxin-fold [9]. This suggests that the mammalian nucleoredoxin-like genes are bi-functional, with one mRNA encoding a cone survival factor and a second encoding an active thioredoxin enzyme involved in the defense against oxidative stress, similar to the situation reported for the RdCVFL homolog in the arthropod *Carcinoscorpius* [12], [13]. The search for proteins targeted by the putative redox-controlling activity of RdCVFL has led to the identification of a functional interaction between RdCVFL and the microtubule association protein TAU [14]. The hyperphosphorylation of TAU in the retina of the mouse carrying a deletion of the *Nxnl1* gene likely plays a role in the degeneration of rods observed in that model and in also in their sensitivity to oxidative stress [15].

A key element in understanding nucleoredoxin-like signaling in the eye is the restricted expression of the *Nxnl1* and *2* genes in the retina. *Nxnl1* expression is fully restricted to the eye, where it is expressed prominently by photoreceptors but also by bipolar cells [8], [10]. Although *Nxnl2* is expressed in selected regions of the brain as well as in the eye [9], within the retina RdCVF2 and RdCVF2L are expressed exclusively by photoreceptor cells. In the *rd1* mouse model of RP, the death of rods (which account for 97% of all photoreceptors) results in the loss of retinal expression of all the products of the nucleoredoxin-like genes, including expression of *Nxnl1* from bipolar cells [10]. This highly regulated expression pattern of the *Nxnl1* and *2* genes raised the interesting question as to what are the mechanisms that control its cellular specificity within the retina. Understanding the regulation of the RdCVFs is not only important for what it can teach us about fundamental questions of cell type-specific gene transcription and processing, but also because manipulating expression of members of the RdCVF family could have therapeutic potential. Work from a number of groups, using a variety of experimental approaches, has identified some of the transcription factors that mediate photoreceptor gene expression. Among the factors identified are CRX, NRL, NR2E3, RX/RAX, PAX6, and QRX [16]–[24]. Interestingly, many of these factors are homeodomain proteins, and it seems that combinatorial use of a relatively small number of proteins can account for a significant degree of photoreceptor specificity. Also of interest, mutation of or modulation of the expression of many of these proteins can cause abnormalities of photoreceptor development and/or retinal degeneration. For example, the homeoprotein CRX (cone rod homeobox), which itself is expressed predominantly in photoreceptors, binds to the consensus sequence C/TTAATCC/A and has functionally important binding sites present in the upstream region of many photoreceptor-specific genes, including rhodopsin, arrestin, IRBP, and β-phosphodiesterase-6 [18], [19], [24]. Knock-out of CRX in mice does not significantly alter photoreceptor lineage decisions, but it does markedly affect photoreceptor differentiation both morphologically and at the gene expression level [25], [26]. In humans, CRX mutations induce a variety of phenotypes of retinal degeneration [27].

In a first step towards understanding the regulation of RdCVF expression, we recently used a combined promoter and transcriptomic analysis to identify some of the transcription factors that modulate *Nxnl1* expression, and among the factors identified were the homeoproteins CHX10/VSX2, VSX1, and PAX4, and the zinc finger protein SP3 [10]. Here, we have extended that analysis and focused on the cis-acting 5′-upstream regulatory sequences in both the murine and human *Nxnl1* and *2* genes. Since a bioinformatic evolutionary comparison analysis (phylogenic footprinting) did not reveal significant sequence conservation between the 5′-regions, we utilized an experimental approach consisting of transient transfection of luciferase reporter constructs in four distinct cell lines and *ex vivo* electroporation of mouse retinal explants with GFP reporter plasmids. The use of the retinal explants allowed analysis of cell type-specific elements. We mapped active proximal promoter regions in both species to small regions of the *Nxnl1* and *Nxnl2* genes. The expression pattern of the electroporated GFP-reporters showed that while the identified *Nxnl1* promoter sequence specifies expression to photoreceptor cells, the identified *Nxnl2* active promoter fragments are not sufficient to restrict reporter expression to photoreceptors. However, we did find that combining a minimal and inactive 134 base pair (bp) piece of the *Nxnl1* promoter with an active 79 bp piece of the *Nxnl2* upstream region could restore photoreceptor specificity. This suggested that the 134 bp *Nxnl1* sequence contains sufficient information for photoreceptor specificity. Furthermore, by mutation analysis we showed that a CRX binding element located within the 134 bp *Nxnl1* fragment is necessary for promoter activity, thereby implicating CRX in the cell type-specific regulation of RdCVF expression.

## Results

### Mapping the transcriptional start sites of the murine and human *NXNL1* and *NXNL2* genes

**Figure 1:**
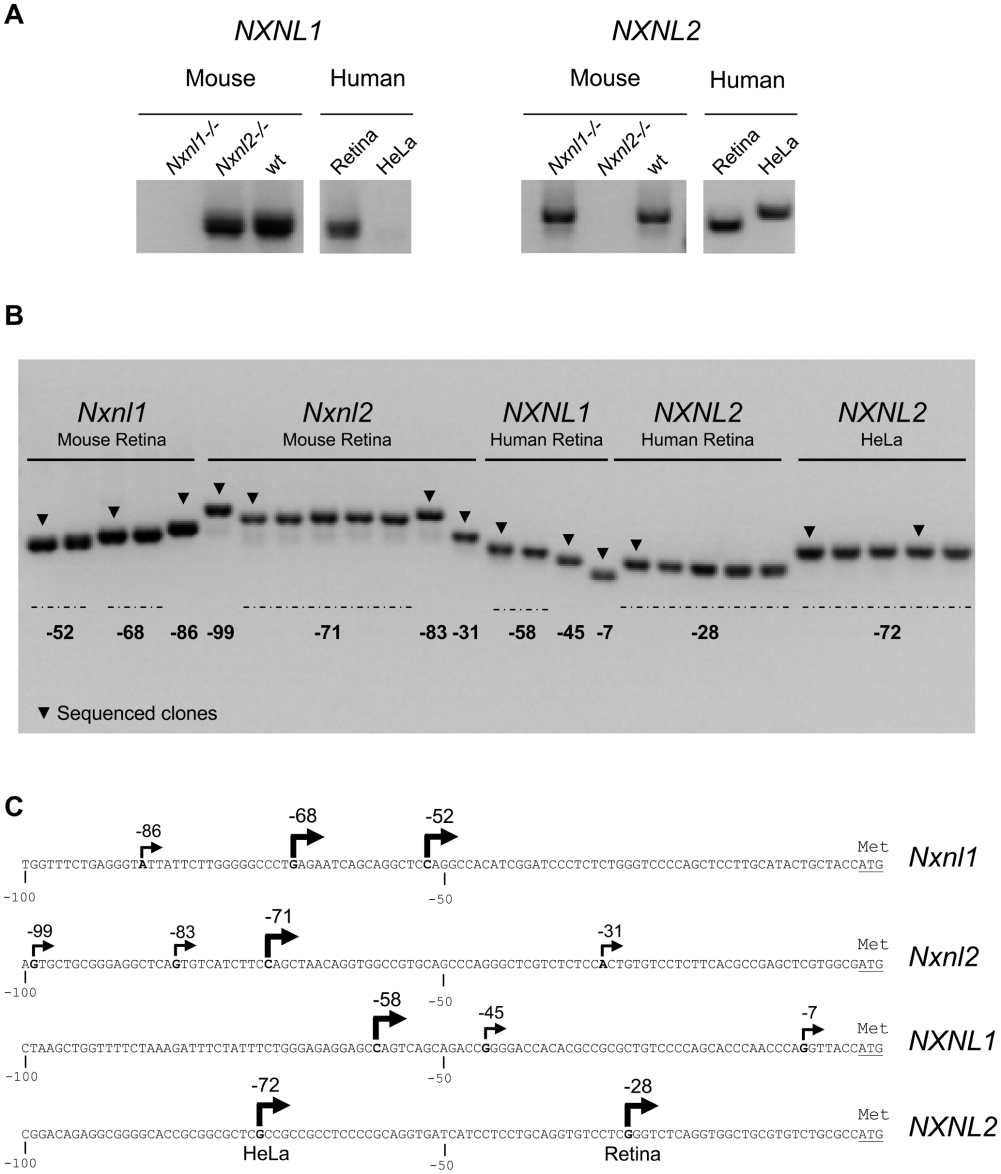
Identification of the putative transcriptional start sites (TSSs) of the *NXNL1* and *NXNL2* genes. (A) Gel electrophoresis analysis of the RACE products. (B) Gel electrophoresis of the cloned RACE products. Arrowheads indicate the products that were sequenced. The numbers indicate the position of the putative TSSs relative to the ATG translation initiation codon. (C) Positions of the putative TSSs mapped onto the genomic sequences of the *NXNL* genes using the ATG as reference (+1). In all subsequent figures and in the text the position of the major putative TSS is defined as +1.

In order to delineate the 5′ upstream regions of the mouse and human *NXNL1* and *NXNL2* genes, we determined the position of their putative transcriptional start sites (TSSs) by rapid amplification of cDNA ends (RACE), using RNA purified from mouse and human retinas. As negative controls, we performed the reactions using RNA isolated from the retina of the *Nxnl1* and *Nxnl2* knock-out mice since the strategy used to generate these animal models results in the elimination of their respective mRNAs [15] and Jaillard et al., (submitted). Additionally, as control for human retinal tissue, we used HeLa cells (ATCC CCL-2) since northern blot analysis showed that *NXNL1* transcripts are not expressed by these cells in Audo et al., (manuscript in preparation). As expected, RACE-PCR products for both *Nxnl1* and *2* were present with wild-type (wt) retina, while with *Nxnl1-/-* RNA only a product for *Nxnl2* was generated, and with *Nxnl2-/-* RNA only a product for *Nxnl1* was generated (Figure 1A). In addition, we confirmed that both *NXNL1* and *NXNL2* are expressed in the human retina and that HeLa cells express *NXNL2* but not *NXNL1*. The products of the RACE reactions were cloned, analyzed by gel electrophoresis and representative clones were sequenced (Figure 1B). Three and four retinal putative TSSs were identified for the mouse *Nxnl1* and *Nxnl2* genes, respectively, three for human retinal *NXNL1*, and only one for human retinal *NXNL2* (Figure 1C). For nomenclature purposes, we have chosen to label the nucleotides in each gene based on the position of its major putative TSS (+1) (since *Nxnl1* had two major TSSs of similar strength, one at −68 bp and one −52 bp, we arbitrarily chose to use the one at −52 bp for labeling). Interestingly, while as noted above *NXNL2* is expressed by HeLa cells, its major putative TSS in HeLa cells is at −72 bp, as compared to −28 bp in the retina, suggesting a difference in the mechanism controlling its expression in photoreceptor versus HeLa cells.

### Murine and human *NXNL1* and *NXNL2* promoter activity in retinal and non-retinal cell lines

In most instances, the major DNA elements regulating promoter activity are located in the 5′-upstream region of a given gene [28]–[30]. In order to map the promoter regions of the murine and human *NXNL1* and *NXNL2* genes, we used PCR to construct a collection of reporter plasmids containing various sequences 5′ to the respective gene's ATG site into the promoterless luciferase reporter vector pGL4.17. The human samples were generated from DNA that was derived from a single donor's white blood cells. DNA sequencing of the resultant constructs, as well as sequencing of additional independent PCR products, and comparison with the UCSC database revealed both previously identified and novel sequence variants within the upstream regions of both the *NXNL1* and *NXNL2* genes (since the term “polymorphism” is generally used to refer to sequence variants that occur at a frequency of at least 1% in a population, and we do not have any frequency data on the variants we identified, in the text below we will refer to these as “sequence variants”). The identity and location of the detected sequence variants and their occurrence within the various reporter constructs studied are noted in the appropriate figure legends. For initial analysis, reporter plasmids were transfected into the Y79 retinoblastoma cell line, which based on the NCBI EST database expresses RdCVF, and into the non-retinal cell line HEK 293, which was derived from adenoviral DNA transformed human embryonic kidney cells [31]. The activity of the various promoter constructs was measured using a dual luciferase assay to normalize expression relative to that of a HSV-thymidine kinase promoter (pRL-TK-renilla), and the data are presented as fold induction compared to the empty pGL4.17 plasmid.

**Figure 2:**
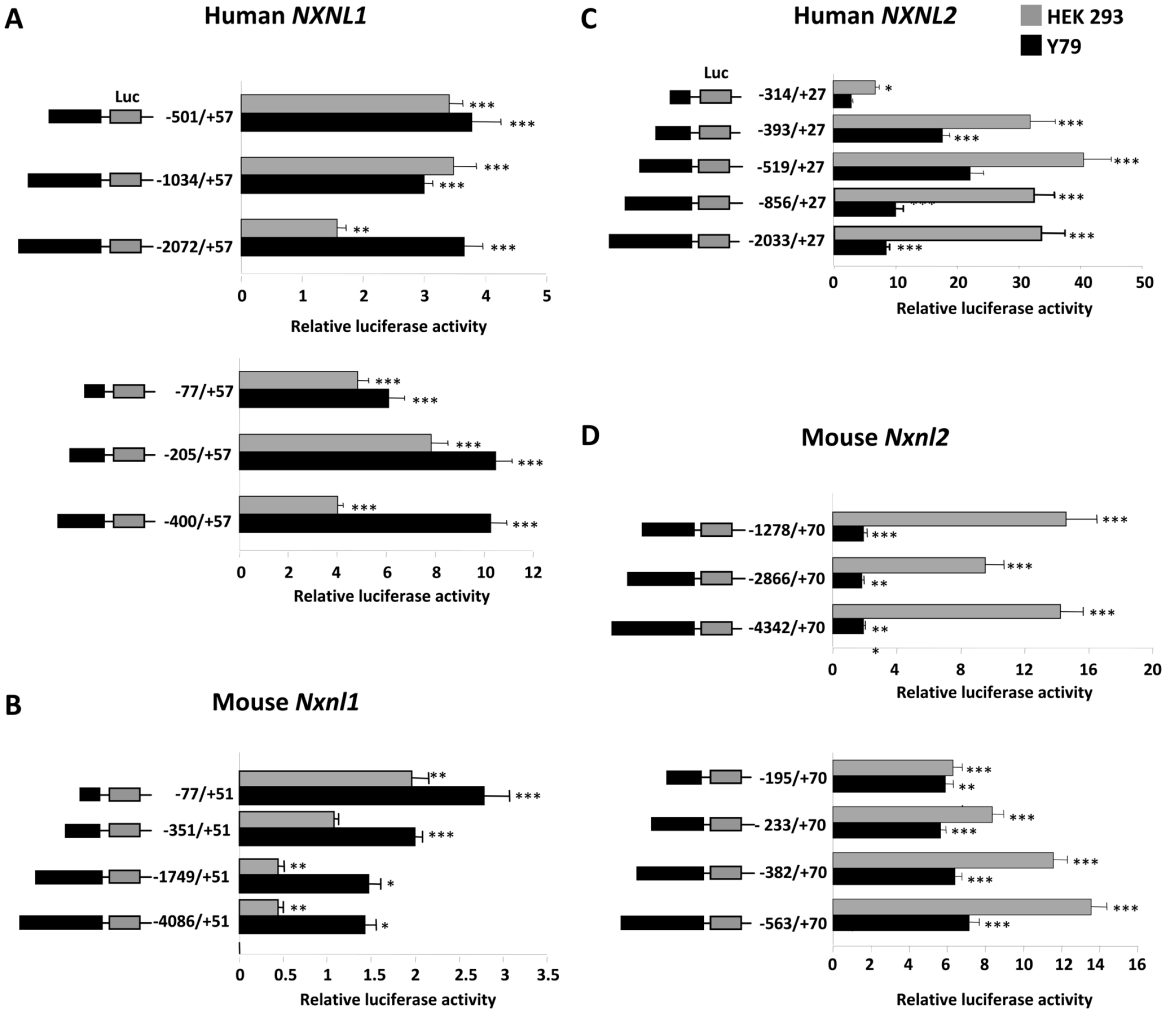
Relative activity of murine, human *NXNL1* and *NXNL2* upstream region promoter fragments in HEK 293 and Y79 cells. (A) Human *NXNL1* constructs. (B) Mouse *Nxnl1* constructs. Sequence positions are numbered with the major putative TSS defined as +1. Luciferase values were normalized to control (pRL-TK) and the results are plotted as fold induction compared to pGL4.17 control plasmid. Values represent the average of at least three independent experiments, each done in triplicate. Error is shown as SEM. **p<0.01, ***p<0.001. The human constructs used in this and subsequent figures contain a C/T sequence variation at position -30 (T is the consensus sequence at this position) and an A/G sequence variation at position -8 (A is the consensus sequence at this position). The −77/+57 construct has a T at position -30 and a G at position -8; the −205/+57 construct has a T at position -30 and G at position -8; the −400/+57 construct has a T at position -30 and A at position -8; the −501/+57 construct has a C at position -30 and A at position -8; the −1034/+57 construct has a T at position -30 and G at position -8; and the −2072/+57 construct has a C at position -30 and A at position -30. (C) Human *NXNL2* constructs. (D) Mouse *Nxnl2* constructs. Luciferase activity was normalized to control (pRL-TK) and the results are plotted as fold induction compared to pGL4.17 control plasmid. Values represent the average of at least three independent experiments done in triplicate. Error is shown as SEM. **p<0.01, ***p<0.001.

With the *NXNL1* transfection series and Y79 retinoblastoma cells, we found similar levels of reporter activity with the −2072 to +57 bp, −1034 to +57 bp, and −501 to +57 bp constructs (Figure 2A). In HEK 293 cells we found similar levels of reporter activity with the −1034 to +57 bp and −501 to +57 bp constructs, but observed significantly less activity with the −2072 to +57 bp construct, suggesting that a negative DNA element(s) active in 293 cells, but not in the retina, might be present in the −2072 to −1034 bp region. Using an analogous approach with a set of shorter deletion constructs, we localized much of the promoter activity to the immediate upstream sequence in Y79 cells, and probably also HEK293 cells, to the region between −205 and −77 bp (Figure 2A). Here again, there is a suggestion of a negative regulatory element specific to HEK 293 cells, this time localized to the −400 to −205 bp region.

In contrast to these findings of significant promoter activity with the human *NXNL1* upstream region, with murine *Nxnl1* we identified only minimal promoter activity with the constructs tested (Figure 2B). Similar results were obtained with two other cell lines, the mouse cone-like line 661W [32] cells and COS-1 cells, derived from African green monkey kidney cells (Figure S1). As noted above, there is surprisingly little sequence conservation between the murine and human *NXNL1* upstream regions. Thus one possible explanation for the mouse constructs lack of significant promoter activity with the monkey and human cells is that they may lack the transcription factors necessary to recognize the murine regulatory elements, and based on the results with mouse retinal explants (see below), this does seem likely to be at least part of the explanation (Figure 2B). However, species difference does not explain the difference observed with the murine 661W cells.

In the case of the *NXNL2* gene, contrary to that observed with *NXNL1*, relative promoter activity was higher in the non-photoreceptor cell line HEK 293 than in Y79 cells (Figure 2C). The major component of the observed positive regulatory activity, in both HEK 293 and Y79, cells was localized to the region between −393 and −315 bp (Figure 2C). As with the human gene, the murine *Nxnl2* promoter constructs were also considerably more active in HEK 293 than in Y79 cells (Figure 2D). The step-wise reduction in activity observed with the shorter deletion constructs in the HEK 293 cells suggests the presence of multiple regulatory elements, each of moderate activity (Figure 2D). Comparable results were obtained with 661W and COS-1 cells (Figure S1).

### Murine and human *NXNL1* and *NXNL2* promoter activity in electroporated mouse retinal explants

**Figure 3:**
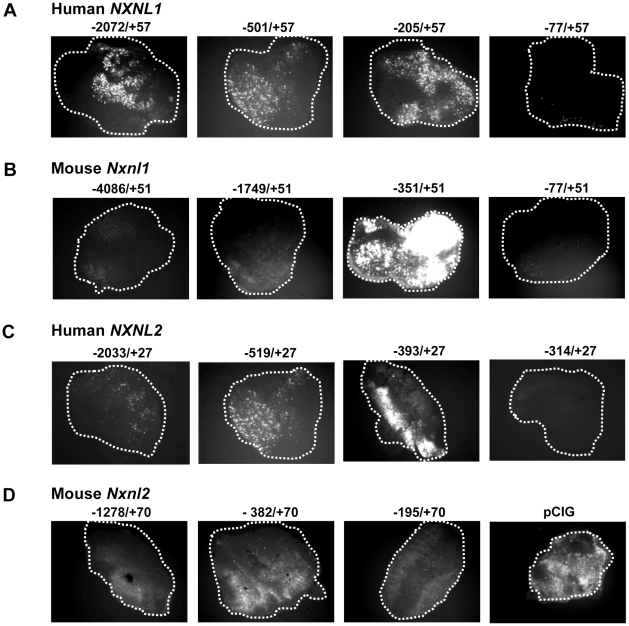
Mapping the active regions of the murine and human *NXNL1 and NXNL2* promoter constructs using electroporation of mouse retinal explants. Retinal explants were electroporated at P0 with the GFP expression vector driven by different fragments of the *Nxnl* promoters and cultured for 5 days in vitro (DIV5). (A) *NXNL1* (B) *Nxnl1* (C) *NXNL2* (D) *Nxnl2*. The intensity of fluorescence of a GFP reporter under the control of the chicken β-actin promoter (pCIG) is provided for comparison.

Based on the lack of clear retinal specificity in the above described cell line studies, which in fact was not surprising given the clear importance of cellular environment on transcriptional activity, we switched to electroporation of perinatal mouse retinal explants, an *ex vivo* approach that we thought would be more amenable to analysis of retinal cell-specific promoter elements. Prior studies by Matsuda and Cepko had shown that mouse retina can be transfected reasonably efficiently by electroporation and that in these experiments retinal promoters tend to retain their cell type specificity [33], [34]. Using an adaptation of their methods, we performed electroporation with various green fluorescent protein (GFP) reporter constructs analogous to the previously described *Nxnl1* and *Nxnl2* luciferase constructs, and then quantified the activity of the promoter fragments by measuring the cellular fluorescence of the electroporated retinas. Mouse retinal explants were electroporated at post-natal day 0 (P0) and maintained in culture 5 days. Fluorescent signals were captured with a Charge Coupled Device (CCD) camera. Using this approach, although there was certainly significant variation from electroporation to electroporation, even with the same construct, we were able to observe fluorescent signals whose intensity was highly dependent upon the upstream sequence contained in the reporter construct. For example, with human *NXNL1*, we observed fairly similar expression levels with the −2072 to +57 bp, −501 to +57 bp, and −205 to +57 bp constructs, but when the upstream region was deleted to −77 to +57 bp there was essentially complete loss of activity (Figure 3A). Although consistent in a general sense with the pattern of the Y79 and HEK 293 results, quantitatively the mouse retina result was significantly different in that with the cell lines the decrease in activity with deletion of the −205 to −77 bp region resulted in only a 40% decrease in activity (Figure 2A). Other differences between the cell line and retinal explant results were even more striking. Unlike with the cell lines, in the mouse explants the murine *Nxnl1* promoter constructs are more active than the human *NXNL1* (compare Figure 2A-B with Figure 3A-B). With the cell lines, the *Nxnl1* construct with the maximal activity was the −77 to +51 bp fragment (Figure 2B), whereas this construct had no detectable activity in the explant system (Figure 3B). The consistently low activity of the −1749/+51 bp construct in the explant system compared to the −924 to +51 bp construct most probably indicates the presence of a negative regulatory element located between −1749 and −924 bp.

With the *NXNL2* promoter constructs, the retinal explant results were fairly consistent with those obtained with the cell lines (compare Figure 2C and Figure 3C). As with the cell lines, the sequence from −393 to −315 bp was required for significant promoter activity. This finding delineates a region of 79 bp (−393 to −315) that presumably contains a critical DNA element(s). The intensity of GFP fluorescence was lower with the longer *NXNL2* constructs (−2033/+27 and −519/+27), suggesting the presence of a negative element in the more far upstream region. Surprisingly, the murine *Nxnl2* constructs tested with the mouse explants showed minimal and inconsistent activity, although it was clear that the −195 to +70 region did have some activity (Figure 3D).

**Figure 4:**
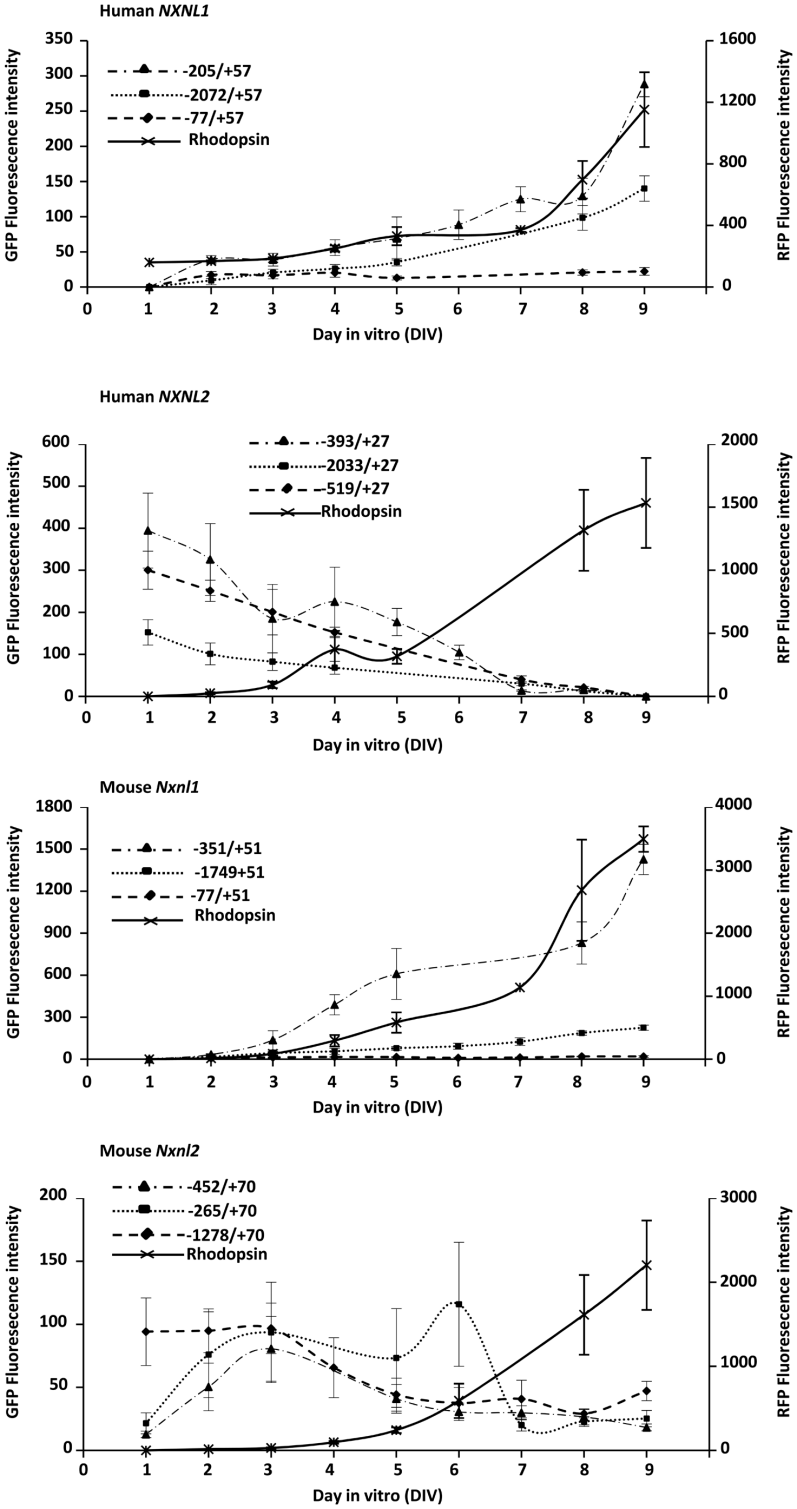
Temporal activity of the murine and human *NXNL1 and NXNL2* promoter constructs in electroporated retinal explants. GFP fluorescence was monitored daily from DIV1 to DIV9. The RFP expression driven by bovine rhodopsin promoter was used as positive control. Quantification of the fluorescence of the retinal explants. GFP expression *NXNL1*, *Nxnl1, NXNL2* and *Nxnl2*, and RFP for rhodopsin at DIV2, 3, 4, 6 and 8. Values represent the average of at least three independent experiments done in triplicate. Error bars represent SEM.

We have previously reported that the temporal pattern of expression of RdCVF and RdCVF2 mRNA in the retina of the mouse shows increasing expression during development that then plateaus when the retina reaches its mature state, a pattern that is fairly similar to that of rhodopsin except that the actual level of expression is considerably lower [8], [9]. As part of our characterization of the various *NXNL1* and *NXNL2* promoter fragments, we performed a time course study with the retina explants, and also tested the bovine rhodopsin 2.2 kb promoter (−2174/+70) as a comparative control [35]. A plasmid containing the −2174/+70 rhodopsin sequence upstream of red fluorescent protein (RFP) was co-electroporated with part of the deletion series of *NXNL1* and *NXNL2* GFP reporter constructs. Fluorescence was monitored daily over a period of 8 days and the intensity of the signal was quantified (Figure 4). As expected, the intensity of RFP fluorescence increased over this time period, corresponding to the postnatal differentiation of the retina. The pattern of expression of the *NXNL1* and *NXNL2* promoters differed temporally from each other in that the *NXNL1* constructs showed a continuous increase in activity with time whereas the *NXNL2* constructs tended to show an early peak, usually at 1 day, and then show a decrease with time. The same regions that showed positive promoter activity in the single time-point studies, −205 to −78 bp and −351 to −78 bp for *NXNL1* and *Nxnl1*, respectively, were found to be important for the observed temporal increase in promoter activity.

### Murine and human *NXNL1* promoter constructs drive expression of GFP specifically in photoreceptors

**Figure 5:**
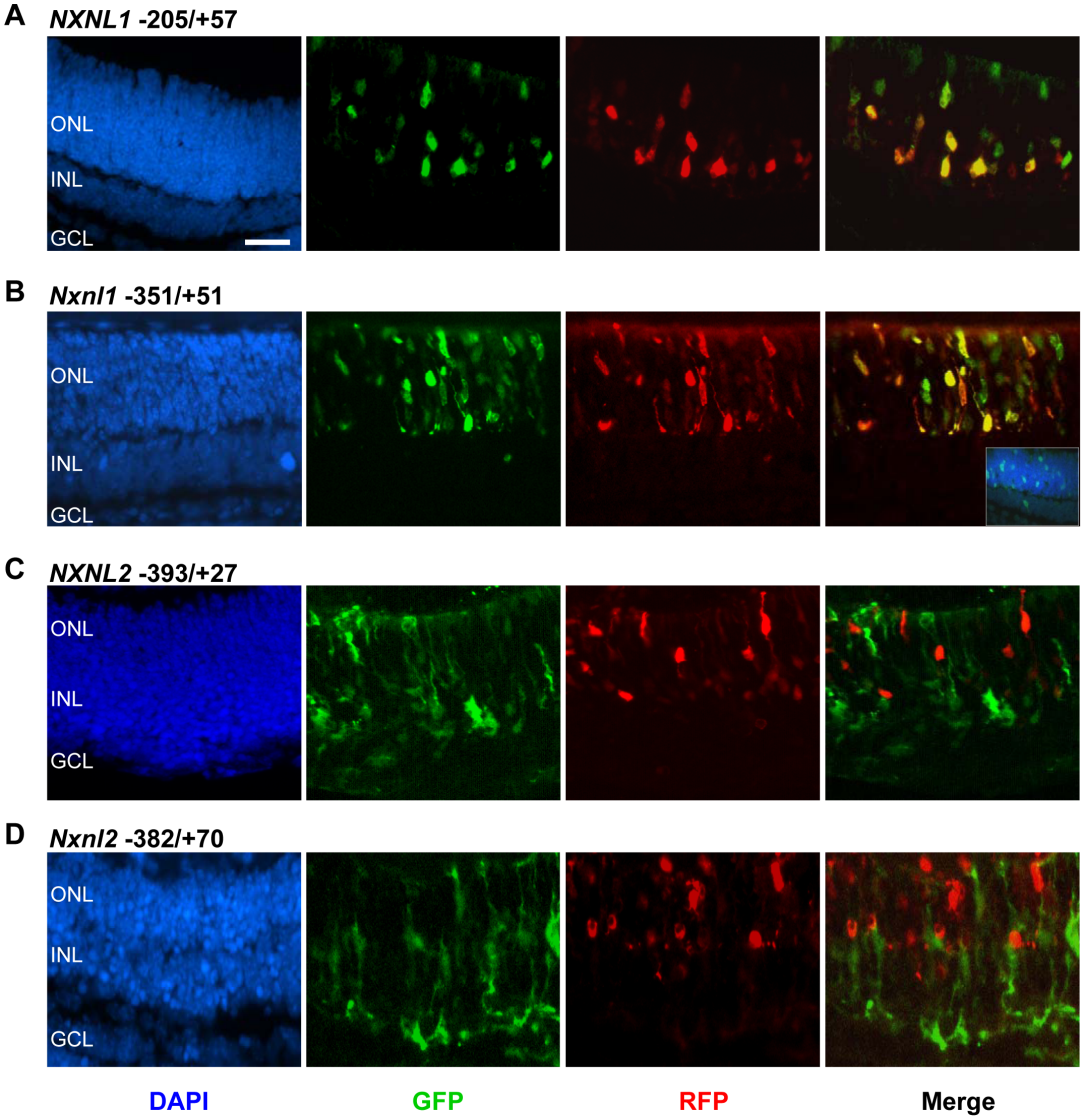
Cell-restricted expression of the murine and human *NXNL1 and NXNL2* promoter constructs. Electroporated retinal explants were sectioned at DIV6, fixed and stained with antibodies against GFP (green, *NXNL* constructs) and RFP (red, rhodopsin construct marking rod photoreceptors). (A) *NXNL1* (B) *Nxnl1* (C) *NXNL2* (D) *Nxnl2*. ONL: outer nuclear layer; INL: inner nuclear layer; and GCL: ganglion cell layer.

We next investigated the cell type specificity of the various promoter regions by determining the spatial distribution of GFP and RFP positive cells in slices of retinal explants 5 days after co-electroporation with the rhodopsin and *NXNL1* and *NXNL2* promoter constructs. With the human *NXNL1* construct, −205/+57 GFP positive cells were observed exclusively in the outer nuclear layer, the part of the retina that corresponds to photoreceptor cell bodies (Figure 5A). We also observed the co-localization of GFP and RFP in a substantial number of cells, indicating that the *NXNL1* promoter construct is active in rods. The construct might also be active in cones as seen by the two GFP-positive/RFP-negative cells at the upper right corner of the panel, but this issue remains to be more definitively resolved. The results were quite similar with the mouse *Nxnl1* construct −351/+51 (Figure 5B), but by examining more extensively the labeled slices, we could identify some GFP positive cells in the inner part of the retina as represented in the merged portion of the panel. These non-photoreceptors cells where the *Nxnl1* promoter is active are likely to be bipolar cells, because endogenous RdCVF mRNA is also expressed by bipolar cells [10]. These observations demonstrate that the −205 to +57 bp human *NXNL1* sequence and the −351 to +51 bp murine *Nxnl1* sequence are each sufficient, at least in retinal explants, to drive the expression of RdCVF specifically in photoreceptors.

The results were strikingly different when retinal explants were electroporated with GFP driven by the mouse and human *NXNL2* promoter 5′-upstream regions. With the human -393/+27 *NXNL2* promoter construct, GFP-positive cells were observed mostly in the inner part of the retina (Figure 5C) - the difference from the rhodopsin promoter can be easily appreciated by the absence of co-localization in the merged image. A similar situation was observed with the mouse *Nxnl2* −382/+70 construct (Figure 5D). The GFP pattern of expression resembles that of Muller glial cells. The absence of expression of these *Nxnl2* reporters in photoreceptor cells cannot be attributed to the lack of transfection of the plasmids into photoreceptors since we could visualize RFP-positive rods in the same retinal explants. Similar results were obtained whatever the length of the upstream regions for both the human and mouse *NXNL2* genes (data not shown), demonstrating that important photoreceptor-specific elements regulating the expression of *Nnxl2* in photoreceptor cells are likely localized outside the 4.5 kb 5′-upstream region that we tested.

### The *NXNL2* -393 to -315 bp region contains a positive transcriptional regulator and the *NXNL1* −77 to +57 bp region contains a photoreceptor-restricting element

**Figure 6:**
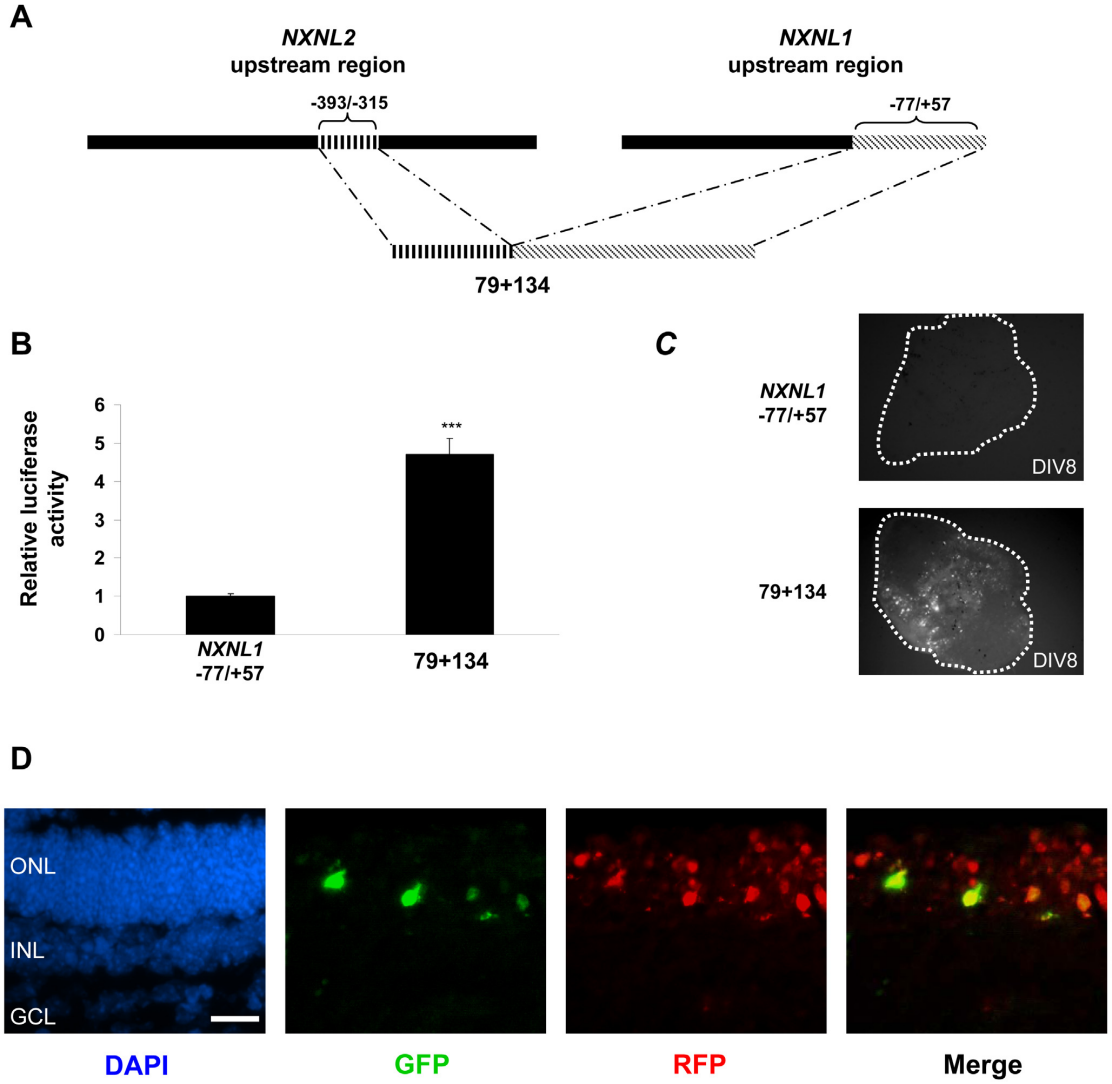
*NXNL1* proximal promoter region, which itself lacks promoter activity, confers photoreceptor-specificity to a *NXNL2* promoter region with positive regulatory activity. (A) Schematic diagram of the 79+134 construct, which consists of the *NXNL1* sequence extending from −77 to +57 bp fused to the *NXNL2* sequence extending from −393 to −315 bp. In terms of the sequence variants mentioned above, it has a C at position -30 and a G at position -8. (B) Transient transfection analysis performed in HEK 293 cells and normalized to control (pRL-TK). The results are expressed as fold induction compared to the *NXNL1* −77/+57 construct. Values represent the average of at least three independent experiments done in triplicate. Error is shown as SEM. (C) Mouse retinal explants electroporated at P0 and maintained in culture for 8 days. (D) Retinal explants were sectioned at DIV8, fixed and stained with anti-GFP (green) and anti-RFP (red) antibodies. ONL: outer nuclear layer; INL: inner nuclear layer; and GCL: ganglion cell layer. ***p<0.001.

Our mapping analysis of the human *NXNL2* promoter indicated the presence of a positive regulatory element located between −393 and −315 bp that is active in the retina but that is not sufficient to provide photoreceptor specificity (Figure 3C and data not shown). To determine whether 79 bp sequence requires other elements of the *NXNL2* promoter not included in the constructs for its activity, we separated it from the rest of the RdCVF2 promoter, by cloning it into pGL4.17 immediately upstream of the *NXNL1* promoter sequence from −77 to +57 bp (79+134, Figure 6A). We chose the −77/+57 *NXNL1* fragment as the scaffold for these experiments because this minimal promoter sequence contains needed core RNA polymerase II binding elements, since it is active in Y79 and HEK 293 cells (Figure 2A), but it is by itself insufficient for transcriptional activity in the more stringent and biologically relevant environment of retinal explants (Figures 3A and 4). When transfected into HEK 293 cells, the 79+134 fusion construct stimulated luciferase activity approximately 5-fold over the basic −77/+57 construct (Figure 6B). When both constructs were tested using the retinal explant assay, the −77/+57 human *NXNL1* construct was found to be inactive as observed earlier (Figures. 3A and 4). However, the 79+134 fusion construct was active, and perhaps surprisingly, its activity was found to be photoreceptor-specific (Figure 6C-D). The merged image confirmed that the GFP-positive cells, which were only observed within the ONL, were also rhodopsin (RFP) expressing cells.

### A CRX element controls the expression of RdCVF and is responsible for its photoreceptor-specific expression

**Figure 7:**
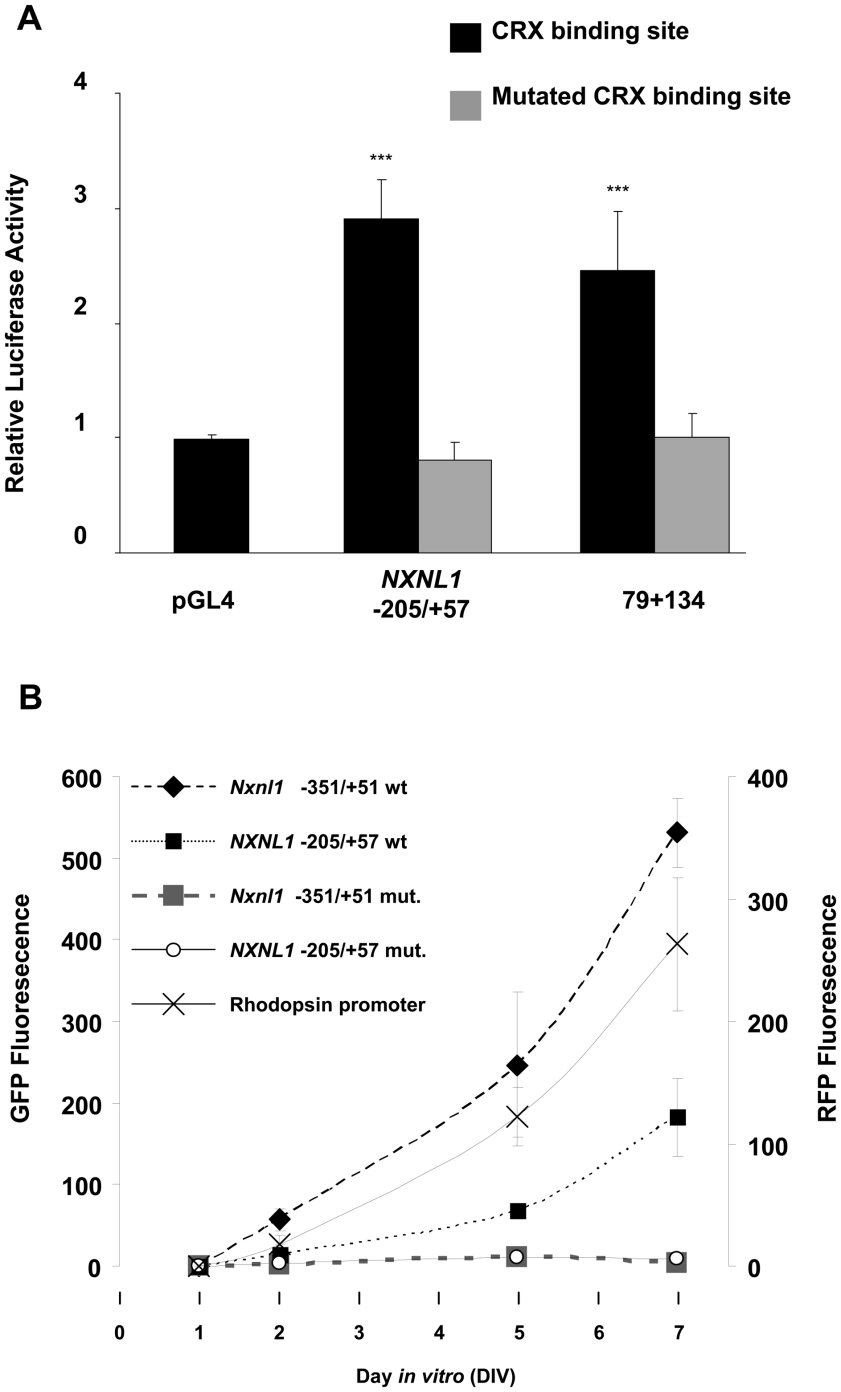
The proximal CRX binding element controls activity of the mouse and human *NXNL1* promoters. (A) Transient transfection analysis performed in Y79 cells with *NXNL1* −205/+57 and the 79+134 *NXNL1*/*NXNL2* fusion construct. The −205/+57 wt and CRX mutant constructs both have a T at position -30 and an A at position -8. Luciferase activity was normalized to control (pRL-TK) and the results displayed as fold induction compared to the pGL4.17 control plasmid. Values represent the average of at least three independent experiments done in triplicate. Error is shown as SEM. ***p<0.001. (B) P0 mouse retinas were electroporated with the RFP rhodopsin expression construct and the indicated GFP construct. Quantified fluorescence intensity of the electroporated retinal explants. Comparison of *NXNL1* −205/+57 wild-type (wt) and CRX mutant constructs (mut). The −205/+57 wt and CRX mutant constructs both have a T at position -30 and an A at position -8. Comparison of *Nxnl1* −351/+51 wild-type (wt) and CRX mutant constructs (mut).

We took a bioinformatics approach, using Genomatix promoter analysis software, to try to identify elements within the *NXNL1* −77/+57 region that could account for its biological activity. The analysis identified four binding elements corresponding to transcription factors reported to be expressed specifically or preferentially in the eye (CRX, MAF, POU1F1, and POU3F4). A similar analysis was performed on the mouse *Nxnl1* −352/+51 promoter sequence and two transcription factors specific to the eye were found to be common between the two analyses: a MAF-response element half site and a putative CRX binding element. Although MAF-response element half sites can show activity, usually less than that observed with full sites [36], we chose to concentrate our efforts on the putative CRX sites, located at position −56 to −50 bp in the human gene and at −54 to −48 bp in the murine gene. The contribution of the identified CRX binding elements to *NXNL1* promoter activity was assessed by mutating the TAA in the TAAT core recognition site to CCG in the human and mouse −205/+57 and −353/+51 promoter constructs, respectively. We choose the CCG mutation because this change has been previously reported to abolish CRX binding to DNA [37]. The wild-type and mutated human constructs were tested in Y79 cells, which express *CRX* as indicated by RT-PCR analysis (result not shown). As expected, the wild-type -205/+57 *NXNL1* construct was active, but when the CRX site was mutated promoter activity dropped to that of the empty luciferase control vector (Figure 7A). We then tested the effect of CRX mutation of the human and murine constructs in a time-course analysis in the retinal explant system (Figure 7B). Mutation of the CRX site led to complete elimination of promoter activity for both the human and murine constructs. These results demonstrate that the proximal CRX binding element present in the human and mouse *Nxnl1* upstream regions is necessary for their promoter activity.

**Figure 8:**
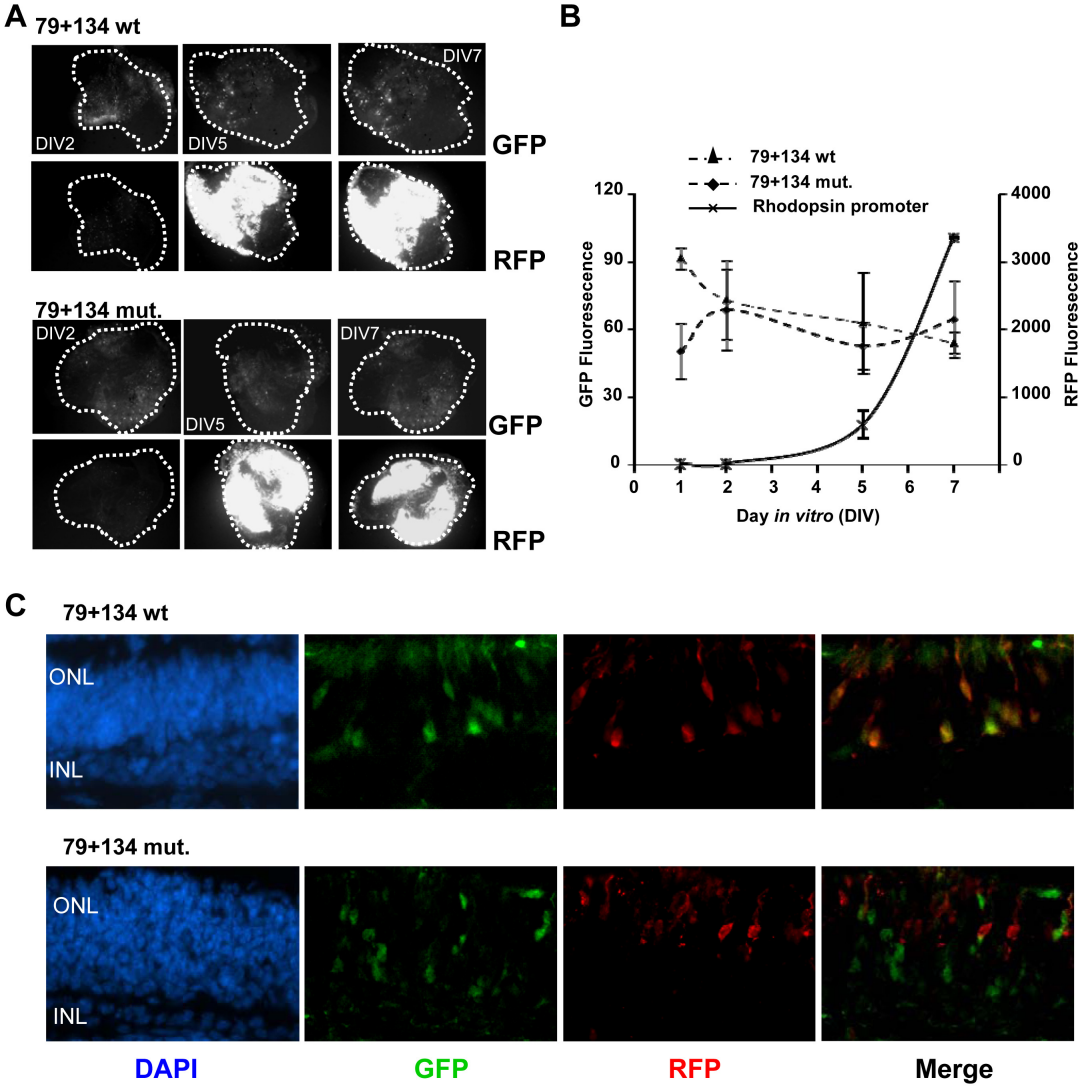
The proximal CRX binding element within the *NXNL1* is important for photoreceptor cell type specificity. P0 mouse retinas were electroporated with the rhodopsin RFP expression construct and the indicated GFP construct. (A) Comparison of 79+134 wild-type (wt) and 79+134 CRX mutant constructs (mut). The 79+134 wt and CRX mutant constructs both have a C at position -30 and a G at position -8. (B) Quantified fluorescence intensity of the electroporated retinal explants. (C) Sectioned retinal explants showing the regional localization of reporter expression in the layers of the retina. ONL: outer nuclear layer and INL: inner nuclear layer. ***p<0.001.

We also tested the effect of the mutation of the proximal CRX binding element in the 79+134 *NXNL2/NXNL1* fusion construct. In the Y79 assay, the *NXNL1* CRX mutation within the fusion construct led to loss of promoter activity, just as observed with the *NXNL1* -205/+57 construct (Figure 7A). When tested in the *ex vivo* retinal explant assay, the CRX mutation in the context of the fusion construct did not lead to loss of promoter activity (Figure 8A-B). However, the CRX mutation did lead to a major change, a loss of photoreceptor specificity, as shown by the presence of multiple GFP positive cells within the inner part of the electroporated retina (Figure 8C).

## Discussion

Our objective in this study was to begin to elucidate the *cis*-elements and *trans*-factors that control the retinal expression of the trophic factors RdCVF and RdCVF2, which are encoded by *NXNL1* and *NXNL2*, respectively. We initially performed transient transfection analysis of a deletion series of murine and human *Nxnl1* and *Nxnl2* 5′-uptream region reporter constructs using both retinal (Y79 and 661W) cell and non-retinal (HEK 293 and COS-1) cell lines. While promoter activity could be detected for a number of the constructs, the absence of evidence of cell-type specificity, i.e. absence of clear and consistent differences in promoter activity for the various constructs between the retinal and non-retinal cell lines, led us to utilize a more physiological assay for assessing retinal promoter activity. Although mouse transgenic studies are considered by many to be the gold standard for examining the cellular and temporal specificity of promoters, such studies are limited by position effects and the by the costs and time involved. As an intermediate approach that combines the practical advantages of cell line based approaches with the cellular specificity of transgenic studies, we adapted the electroporation of mouse retinal explants to provide rapid and reproducible quantitation of cell-specific promoter activity. Significantly, we found that regulatory regions identified, using the same promoter constructs, differed depending upon whether we performed the assays with the cell lines versus the retinal explants, with the explants giving stronger evidence of cell-type specificity. For example, with the Y79 cell assay the *NXNL1* -77 to +57 bp construct retained considerable activity compared to the −205 to +57 bp construct, while in the more stringent explant system the 5′ deletion from −205 to −77 bp led to loss of all detectable promoter activity, indicating that the deleted region contained a sequence with regulatory function that was essential in retinal cells but not in the cell lines tested. This suggests that the activity of the *Nxnl1* promoter, like that of other genes that show highly cell-type restricted expression patterns, is regulated by transcription factors that are sensitive to the cellular context of the cell. Of even greater significance, as demonstrated with the *NXNL1* and *NXNL1/*2 fusion gene studies, the explant system allows analysis of cell type specificity within the retina. Immortalized cell lines are appropriate and provide a powerful system to reveal potent *cis-*acting elements with broad cellular spectrum, and also can be useful for gain of function studies in which non-expressed cell type-specific *trans*-acting factors are added to the cells, but are of limited use for revealing the promoter elements responsible for conferring highly cell-restricted patterns of expression.

The deletion series of the *NXNL1* promoter constructs revealed a critical region proximal to the transcriptional start site of both the human (−205 to −77 bp) and mouse (−351 to −77 bp) genes (Figure 3A and B). Within these regions, *cis* elements are able to confer promoter activity to the reporter constructs, and within the context of more downstream sequences to restrict expression to the naturally RdCVF expressing cells, photoreceptor cells and, to a lesser extent, bipolar cells (Figure 5A and B). The promoter activity scored for these constructs (*NXNL1* −205/+87 and *Nxnl1* −351/+51) seems to depend on the state of differentiation of the retinal cells, and mostly of the differentiation state of photoreceptors since the activity gradually increases in concert with the activity of the rhodopsin promoter construct with time (Figure 4). The bioinformatic analysis of the shortest active *NXNL1* promoter constructs revealed the existence of a putative CRX binding element (−56 to −50 bp in *NXNL1* and −54 to −48 bp in *Nxnl1*). Because of the essential role of the homeodomain protein CRX in the maturation of photoreceptors and its implication in inherited photoreceptor degeneration [18], [38], [39], [25], the role of this binding element was further analyzed by mutagenesis. The integrity of the CRX binding element was found to be essential for the activity of the *Nxnl1* promoter *in vitro* and *ex vivo* (Figures 7A and B). While we cannot rule out the possibility that another promiscuous homeodomain protein regulates the expression of RdCVF through this “CRX” element, CRX it is the most likely candidate to be mediating the observed biological activity.

The deletion analysis of the human *NXNL2* gene identified a 79 bp promoter element that showed promoter activity in both the cell line and retinal explant assay systems (Figures 2C and 3C). The activity of this element is not restricted to photoreceptor cells (Figure 5C). Interestingly, this 79 bp sequence is active when fused to an inactive 134 bp *NXNL1* minimal proximal promoter sequence (Figure 6B and C), resulting in a reporter expression pattern that shows restriction to photoreceptors (Figure 6D). These findings demonstrate that the *NXNL2* 79 bp sequence from −393 to −315 bp contains positive regulatory activity that can act independently of its own basal promoter and also strongly suggest that the *NXNL1* −77 to +57 bp proximal promoter region contains sufficient cell type-specific information, when supplemented with a less specific transcriptional activator, to restrict expression to photoreceptor cells. The identity of the biologically active regulatory elements within the 79 bp *NXNL2* sequence, and the transcription factor(s) that bind to them, are presently unknown. For the cell type restricting activity of the 134 bp *NXNL1* region, the putative CRX binding site discussed above appears to be important. Mutation of the CRX element abolished promoter activity of the *NXNL2*/*NXNL1* fusion construct in Y79 cells (Figure 7A). Although mutation of the CRX site did not significantly reduce promoter activity in the explant system, it did lead to loss of photoreceptor specificity (Figure 8). These results, taken together, suggest that the CRX site within the *NXNL1* proximal promoter region plays a dual function depending on its context. Within the native *NXNL1* promoter it appears to act as a positive transcriptional regulator whose activity is essential for expression. Without its activity there is no detectable expression so in this context its possible role in influencing cell type specificity cannot be assessed. In the context of the *NXNL2*/*NXNL1* fusion construct, presumably because an element, or elements, within the 79 bp *NXNL2* sequence is able to replace its transcriptional activating role, its function in restricting expression to photoreceptors is unmasked. Future studies will hopefully provide mechanistic insight into how CRX carries out these dual roles, and also provide information on the other regulatory sites and factors responsible for the photoreceptor expression of RdCVF and RdCVF2.

## Materials and Methods

### Animals

No animal experimentation was performed. The animals used in this study were obtained from Charles River France (L'Arbresle). Retinal tissues were obtained from newborn C57BL/6 mice. Animals were sacrificed the day of birth and eyes were dissected to recover neural retinal tissues (Authorization 75–1208 delivered on July 21 2008 by the Minister of Agriculture). The methods used to secure animals and tissues complied with the ARVO Statement fro the Use of Animals in Ophthalmic and Vision Research. No animal experimentation was performed. The animals used in this study were obtained from Charles River France (L'Arbresle).

### Rapid Amplification of cDNA Ends (5′ RACE)

The rapid amplification of cDNA ends (5′ RACE) was carried out using total RNA purified from mouse retina, human retina and cultured HeLa cells by CsCl_2_ ultracentrifugation. A FirstChoice™ RLM-RACE Kit (Ambion) was used to identify transcription start sites according to manufacturer's instructions. Briefly, total RNA was treated with calf intestinal phosphatase to remove the 5′-phosphate. The RNA was then treated with tobacco acid pyrophosphatase to remove the cap structure of the mRNA. A synthetic RNA adapter was then ligated to the RNA population to allow ligation to only RNA having been capped. After reverse transcription reaction, cDNA was obtained by nested-PCR using two reverse primers for each gene (human *NXNL1*: 5′ CCGCCCGCAGTACATAGAACTCA 3′ and 5′ TCTGTGAGCCGCACGAAGAAGTC 3′; mouse *Nxnl1*: 5′ TCAGCACGTCCCCACCAG GCTTAA 3′ and 5′ TCAGCACGTCCCCACCAGGCTTAA 3′; human *NXNL2*: 5′ GCGCATGAAGTCCAGCATCTCC 3′ and 5′ TCGGCTGACACGAAGACCACTTC 3′; mouse *Nxnl2*: 5′ CGTTCTGCTTGATGACCACCAGC 3′ and 5′ GGGGATGGCGGTGAT TTCGTA 3′) and the two forward primers provided with the kit. PCR products were analyzed on 2% agarose gels and the reaction products were cloned into pGEM T Easy (Promega). A subset of recombinant clones were analyzed on agarose gel and the plasmid displaying different insert sizes were sequenced from both directions. Theses sequences were aligned to the genomic sequences.

### Cell culture

Human Y79 cells from an immortalized cell line derived from a human retinoblastoma (ATCC HTB-18) were cultured in RPMI 1640 medium supplemented with 10% fetal bovine serum. Cells were maintained in suspension or for transfection purposes were grown as a monolayer. For cell attachment, plates were treated with poly (D-lysine) (0.2 mg/ml, Sigma) and laminin (0.1 mg/ml, Sigma) for 3 hours at 37°C. Human HEK 293 cells (ATCC CRL-1573) and COS-1 cells (ATCC CRL-1650) were cultured in Dulbecco's modified Eagle's medium (DMEM) supplemented with 10% fetal bovine serum (FBS, Invitrogen). 661W cells (murine cone photoreceptor derived cells) were cultured in Dulbecco's modified Eagle's medium (DMEM) supplemented with 10% fetal bovine serum (FBS, Invitrogen) and 2mM L-glutamine (Invitrogen). HeLa cells (ATCC CCL-2) were cultured in RPMI 1640 medium supplemented with 10% fetal bovine serum. Cell media were changed every 2–3 days, and cells were split weekly.

### Promoter reporter plasmids construction

The sequence upstream ATG of both murine and human *Nxnl1* and *Nxnl2* were cloned in the promotorless pGL4.17 (expressing a firefly luciferase, Promega) and peGFP (Clontech) plasmid using the gateway technology (Invitrogen). The vectors were converted into a gateway destination vector by introducing a cassette containing the *att*R sites flanking the *ccd*B gene into the multiple cloning site. To identify DNA sequences that regulate murine and human *Nxnl1* and *Nxnl2* transcription, a series of deletions from the 5′ end of each gene was created. Primers were designed to amplify different lengths of the 5′end regions of each gene, from approximately 100 base pairs (bp) to 4.5 kb and the *att*B sites were incorporated into the forward (*att*B site 5′ GGG GAC AAG TTT GTA CAA AAA AGC AGG CTT A 3′) and the reverse (*att*B site 5′ GGG GAC CAC TTT GTA CAA GAA AGC TGG GT 3′) primers. These primers introduced a TA at the 5′-end of each construct except the CRX mutant and wild-type constructs (see below). The PCR reactions were performed using murine and human genomic DNA as the template and were subjected to a hot start of 5 min at 94°C prior to the addition of the Platinium *Taq* DNA Polymerase High Fidelity (Invitrogen) followed by 7 cycles amplification: denaturing for 25 sec at 94°C, annealing for 30 sec to 7 min at 66°C (depending on the size of the PCR product), 35 cycles amplification: denaturing for 25 sec at 94°C, annealing for 30 sec to 7 min at 60°C (depending on the size of the PCR product) and a final extension at 66°C for 10 min. The primers used for each construct were listed in Table S1. The PCR products were purified after gel agarose electrophoresis using the gel extraction kit (Qiagen). The BP reaction was performed using 300 ng of the PCR product, 300 ng of the pDONR vector (Invitrogen), 4 µl of the BP Clonase Reaction Buffer (Invitrogen) and 4 µl BP Clonase enzyme mix (Invitrogen) to obtain entry clones. The reaction was incubated one hour at room temperature. OneShot TOP10 chemically competent *E.coli* cells (Invitrogen) were transformed by using 5 µl of the BP reaction. Positive clones were identified by restriction enzyme analysis of purified plasmid and verified by DNA sequencing. The LR reaction was performed by using 300 ng of the entry clone, 300 ng of the destination vector (pGL4.17 or peGFP), 4 µl of LR Clonase Reaction Buffer (Invitrogen) and 4 µl of the LR Clonase enzyme (Invitrogen) to obtain the expression vectors. The reaction was incubated one hour at room temperature. OneShot TOP10 chemically competent *E.coli* cells (Invitrogen) were transformed by using 5 µl of the LR reaction. Positive clones were identified by restriction enzyme analysis.

### Construction of mutated CRX binding site

Plasmids containing a mutated CRX binding site (the TAA in the TAAT core recognition site was modified to CCG) were generated by DNA synthesis (DNA2.0) and cloned in pDONR. The LR reaction was performed as previously described to subclone the sequences into pGL4.17 and peGFP. For the synthesis, the consensus sequence from GenBank (NCBI) was used. Because this sequence contained some sequence variants from the above described PCR generated constructs (due to both sequence variations in the donor DNA sample and to nucleotide differences in the PCR primer sequences) and because for the CRX mutation analysis we wanted the mutant constructs to differ from the wild-type constructs only in at the CRX binding site, we used the synthesized constructs as templates to generate wild-type constructs that were indeed identical except at the CRX site. A ‘mutagenic’ primer was used to convert the CCG sequence back to the wild type CRX binding site (TAA) by PCR-based mutagenesis. The resulting fragment was then used as a mega-primer to amplify the full-length construct in a second round of PCR amplification. The full-length wild-type constructs were then cloned back into pDONR on an AvaI –EcoRV fragment, and the resulting plasmids were sequenced. Reporter constructs were generated by Gateway recombination as described above.

### RNA extraction and RT-PCR

Total RNA was extracted from Y79 cells using the RNeasy extraction kit (Qiagen). One microgram of total RNA was reverse transcribed into cDNA by incubation with 1 µl (500 µM final) of deoxyribonucleoside triphosphate solution (dNTP), 1 µl of oligodT (10 µM), 4 µl 5× reaction buffer, 2 µl of 0.1 M dithiotreitol (DTT), 1 µl of RNasin an 200 U of superscript II (Invitrogen) to a final volume of 20 µl. Samples were incubated 1 hour at 37°C and the enzyme was inactivated at 72°C during 10 min. The resulting cDNA were amplified in PCRs using Platinium *Taq* High Fidelity (Invitrogen) in 2 mM MgSO_4,_ 200 µM dNTP and 50 pmol of primers. Oligonucleotides used were: human *CRX* forward: 5′ GGC TCT GAA GAT CAA TCT GCC TGA 3′, reverse: 5′ CCA TAG CTC TGG CCT GAT AGG GAG 3′: human *G6PDH* forward: 5′ ATG TTCGTC ATG GGT GTG AAC C 3′, reverse: 5′ AGG GAT GAT GTT CTT CTG GAG AGC C 3′. Cycling protocol was: 94°C, 2 min; 35 cycles of 94°C, 30 sec; 60°C, 30 sec; 72°C, 2 min, followed by a final extension step at 72°C for 5 min. PCR products were run on a 1.5% agarose gel stained with ethidium bromide and visualized under UV illumination.

### Transient transfection and reporter gene assay

Luciferase activity was determined using a luciferase assay system (Promega) according to the manufacturer's protocol. Briefly, Y79 and HEK 293 cells were plated for 24 h at respectively 5×10^5^ and 2×10^5^ cells/well in 24-well plates. Five hundred (for Y79) or 100 ng (for HEK 293) of promoter constructs or the empty vector (pGL4.17) was transiently transfected into the cells with Lipofectamine 2000 (Invitrogen). Cells were co-transfected with pRL-TK containing *Renilla* luciferase gene driven by the thymidine kinase promoter (Promega). One day after transfection, cell extract was lysed, and the luciferase activity of the extract was measured with the luminometer LB-96V (Berthold). Firefly luciferase activities were normalized by *Renilla* luciferase activities and relative luciferase activities were expressed as fold induction as compared to the empty vector (pGL4.17).

### Electroporation of retinal explants

Mouse retinas from newborns were electroporated the day of birth as described in Matsuda & Cepko [33], [40]. Briefly, newborn retinas were dissected in Hank's Balance Sodium Salts (HBSS) without calcium and magnesium (Invitrogen) and were cut into 3 pieces and were submerged in a 1 mg/ml DNA solution. They were subjected to 5 electric pulses at 25 V with 50 ms duration and 500 ms intervals using a BTX ECM830 square wave generator (BTX). Explants were then placed on polycarbonate filter discs (Dutscher) and cultured in DMEM/F12 medium (Invitrogen) with 10 mM HEPES pH 7.0 containing 5% FBS (Invitrogen). Cultures were maintained up to 9 days and media were changed every 2–3 days. The empty vector pCIG-eGFP used as control expresses the eGFP under the control of the chicken β-actin promoter [40].

### Image acquisition

The fluorescence of retinal explants was monitored after electroporation. Fluorescent signals were captured with a macroscope Z6 APO (Leica) equipped with a CCD CoolSnap camera (Roper Scientific). MetaMorph software (Universal Imaging Corporation) was used to quantify the intensity of the emitted fluorescence

### Immunofluorescence

Retinal explants were fixed with 4% paraformaldehyde (PAF) in PBS for 10 min and incubated 30 min in 30% sucrose at room temperature. Retinal explants were frozen in PBS-10% sucrose-7.5% gelatin using a immersion in dry ice and isopentane. The samples were cut into 10 µm thick sections and mounted on glass slides. To reduce non-specific labeling, the sections were incubated for 2 h at room temperature in blocking solution (PBS, 0.1% Triton X-100 and 2% goat serum). The sections were incubated with the primary GFP- (Roche) and RFP- (Mbl) antibodies in blocking solution (respectively 1∶400 and 1∶2000) for 1 h at room temperature. Sections were washed three times in PBS and were then incubated with 4',6-diamidino-2-phenylindole (DAPI) and secondary antibodies (goat anti-rabbit AlexaFluor 594 and goat anti-mouse AlexaFluor 488, Invitrogen) diluted at 1∶500 in PBS for 1 h at room temperature. After three washes in PBS, immunolabeled sections were mounted with Gel Mount (Biomedia) and were examined with a DM 5000 microscope (Leica) equipped with a CCD CoolSnap camera (Roper Scientific).

### Statistical Analysis

All experiments were performed at least three times, each in triplicate. Data are expressed as the means ± S.E.M. Comparison of means was performed using test. p<0.05 is considered significant.

## Supporting Information

Table S1 Oligonucleotide PCR primers used to generate the various human and mouse *NXNL1* and *NXNL2* promoter constructs.
(0.03 MB DOC)

Figure S1 Relative activity of murine, human *NXNL1* and *NXNL2* upstream region promoter fragments in COS-1 and 661W cells. (A) Human *NXNL1* constructs. (B) Mouse *Nxnl1* constructs. The nucleotide positions of each construct are given in reference to the major putative TSS for each gene. Luciferase activity was normalized to control (pRL-TK) and the results are plotted as fold induction compared to pGL4.17 control plasmid. Values represent the average of at least three independent experiments done in triplicate. Error is shown as SEM. **p<0.01, ***p<0.001. (C) Human *NXNL2* constructs. (D) Mouse *Nxnl2* constructs. The nucleotide positions of each construct are given in reference to the major putative TSS for each gene. Luciferase activity was normalized to control (pRL-TK) and the results are plotted as fold induction compared to pGL4.17 control plasmid. Values represent the average of at least three independent experiments done in triplicate. Error is shown as SEM. **p<0.01, ***p<0.001.
(5.06 MB TIF)
