# Supplementary material for: Expression of Rod-Derived Cone Viability Factor: Dual Role of CRX in Regulating Promoter Activity and Cell-Type Specificity
Source: PLoS One. 2010 Oct 7;5(10):e13075. doi: 10.1371/journal.pone.0013075 (PMC2951342; doi:10.1371/journal.pone.0013075)
Supplement: Table S1 — Oligonucleotide PCR primers used to generate the various human and mouse NXNL1 and NXNL2 promoter constructs. (0.03 MB DOC) [file pone.0013075.s001.doc]

TABLE S1

| **Promoter constructs** | **Primer sequence (5’ to 3’)** |
| --- | --- |
| *NXNL1*-77/+57  *NXNL1*-205/+57  *NXNL1*-400/+57  *NXNL1*-501/+57  *NXNL1*-1034/+57  *NXNL1*-2072/+57 | Reverse GGC GGT AAC CTG GGT TGG GTG CTG GGG AC  Forward CCC TCA GGT CAA GGT TTA GGC AGA TTA GT  Forward TGC CTC TCA AAG TGC TGG GAT CAC AGG CG  Forward GGC ACA ATC TCA GCT CAC TGC AAC CTC CG  Forward GAG TCA CTG TCT GCT ACA TGG TTT TTT GG  Forward GTA CTT TTA GTA GAG ACG GGG TTT CAC TG  Forward CCT TGA AGT AAC TAG TAG AGT CCA TGT GA |
| *Nxnl1*-77/+51  *Nxnl1*-351/+51  *Nxnl1*-1749/+51  *Nxnl1*-4086/+51 | Reverse GGG TAG CAG TAT GCA AGG AGC TG  Forward GTC AGC AGG AGG ATC TAA CCA GGC TAA TC  Forward CTG TTG TGG ACA GCG GAA GCC AGG CCC TG  Forward CCC TTC TCC TAG TTT ATA GAG TGC TAA GT  Forward GAG CAC ACA GCT GAG ACC GCA GGT ACC GC |
| *NXNL2*-314/+27  *NXNL2*-393/+27  *NXNL2*-519/+27  *NXNL2*-856/+27  *NXNL2*-2033/+27 | Reverse GGC GCA GAC ACG CAG CCA CCT GAG ACC C  Forward GAG GGC GCA GGA GTG TGG GCG GGG CGC A  Forward GCA GGG GGT GGA GAG TGC GGA ATG TGG G  Forward AGA GTG TAG GGA TGT GGG CCC AGC GCA G  Forward GGC TGG ACA ACC AGT GGG AGA GGG CGC G  Forward ACT TAG GGG AGA GTA ACT GAT TTT TAG GA |
| *Nxnl2*-195/+70  *Nxnl2*-233/+70  *Nxnl2*-382/+70  *Nxnl2*-563/+70  *Nxnl2*-1278/+70  *Nxnl2*-2866/+70  *Nxnl2*-4342/+70 | Reverse CGC CAC GAG CTC GGC GTG AAG AGG AC  Forward GAT AAA ATA GAG GGT GGG AGA GGT TGA T  Forward GTT AAG GAC TCT GGT GGG TAG AGA GGG TT  Forward GTG GAT TAG GTG GTG TGG AAC TGG GCC CG  Forward AGG AAA GCT GTA AGG CTT ATC TAG GGC TG  Forward CTA AGT TTC AAT AAT TTC CTA CAA CCT TTT  Forward CTC AAA GCA GTT TAT TCA GGA ATC TTT CA  Forward TGG GAG GAG AGG ATG CTA GAG AGG GAA A |
